# High Amounts of S100-Alarmins Confer Antimicrobial Activity on Human Breast Milk Targeting Pathogens Relevant in Neonatal Sepsis

**DOI:** 10.3389/fimmu.2017.01822

**Published:** 2017-12-13

**Authors:** Sabine Pirr, Manuela Richter, Beate Fehlhaber, Julia Pagel, Christoph Härtel, Johannes Roth, Thomas Vogl, Dorothee Viemann

**Affiliations:** ^1^Department of Pediatric Pneumology, Allergy and Neonatology, Hannover Medical School, Hannover, Germany; ^2^Children’s Hospital “Auf der Bult”, Hannover, Germany; ^3^Department of Pediatrics, University of Lübeck, Lübeck, Germany; ^4^Institute of Immunology, University of Münster, Münster, Germany

**Keywords:** neonate, sepsis, S100A8/A9, alarmins, breast milk

## Abstract

Sepsis is a leading cause of perinatal mortality worldwide. Breast milk (BM) feeding is protective against neonatal sepsis, but the molecular mechanisms remain unexplained. Despite various supplementations with potential bioactive components from BM formula feeding cannot protect from sepsis. S100-alarmins are important immunoregulators in newborns preventing excessive inflammation. At high concentrations, the S100A8/A9 protein complex also has antimicrobial properties due to its metal ion chelation capacity. To assess whether BM contains S100-alarmins that might mediate the sepsis-protective effect of BM 97 human BM samples stratified for gestational age, mode of delivery and sampling after birth were collected and analyzed. S100A8/A9 levels were massively elevated after birth (*p* < 0.0005). They slowly decreased during the first month of life, then reaching levels comparable to normal values in adult serum. The concentration of S100A8/A9 in BM was significantly higher after term compared with preterm birth (extremely preterm, *p* < 0.005; moderate preterm, *p* < 0.05) and after vaginal delivery compared with cesarean section (*p* < 0.0005). In newborn *s100a9^−/−^* mice, enterally supplied S100-alarmins could be retrieved systemically in the plasma. To explore the antimicrobial activity against common causal pathogens of neonatal sepsis, purified S100-alarmins and unmodified as well as S100A8/A9-depleted BM were used in growth inhibition tests. The high amount of S100A8/A9 proved to be an important mediator of the antimicrobial activity of BM, especially inhibiting the growth of manganese (Mn) sensitive bacteria such as *Staphylococcus aureus* (*p* < 0.00005) and group B streptococci (*p* < 0.005). Depletion of S100A8/A9 significantly reduced this effect (*p* < 0.05, respectively). The growth of *Escherichia coli* was also inhibited by BM (*p* < 0.00005) as well as by S100A8/A9 in culture assays (*p* < 0.05). But its growth in BM remained unaffected by the removal of S100A8/A9 and was neither dependent on Mn suggesting that the antimicrobial effects of S100A8/A9 in BM are primarily mediated by its Mn chelating capacity. In summary, the enteral supply of bioavailable, antimicrobially active amounts of S100-alarmins might be a promising option to protect newborns at high risk from infections and sepsis.

## Introduction

Breastfeeding and human milk represent the gold standard for infant feeding and nutrition (1). Clinical and epidemiological studies indicate the short- and long-term medical and developmental advantages of breastfeeding compared with formula feeding, i.e., lower incidence of infectious and inflammatory diseases, reduction of the risk for asthma, allergy, and obesity as well as promotion of gastrointestinal, immune, and neurologic development (1–3). Although immunoregulatory, antimicrobial, and antiviral activities have been linked to diverse bioactive components (2, 4, 5), our knowledge on the molecular mechanisms that confer these properties on breast milk (BM) are still very fragmentary.

Newborn infants, especially preterm babies, are at high risk for fatal sepsis characterized by rapid courses with hyperinflammatory immune responses (6). Sepsis is still a leading cause of death among neonates worldwide (7–9). BM feeding significantly decreases the incidence of sepsis in newborns (10–12). However, most efforts of nutritional supplementation of formula with bioactive components from human BM remained without success with respect to decreasing the incidence of infection and inflammation (2, 13).

We previously showed that the endogenous toll-like receptor 4 ligands S100A8 and S100A9 (also known as MRP8 and MRP14 or calprotectin) have an important role in newborns by regulating inflammatory responses (14–16). Activated myeloid cells release heterodimeric complexes of S100A8 and S100A9 called calprotectin (S100A8/A9) (17). Reduced release of these S100-alarmins at birth is associated with an increased risk of newborns to suffer from sepsis (15). Systemic application of S100-alarmins protects neonatal *s100a9*^−/−^ mice from hyperinflammation and fatal courses of sepsis (15, 16). Next to these immunoregulatory effects on innate immune responses, inhibiting as well as promoting effects on the growth of bacteria have been ascribed to S100A8/A9 (18–21). Growth inhibition of bacteria by S100A8/A9 has been shown for *Staphylococcus aureus* and *Klebsiella pneumoniae*. This effect has been attributed to the metal ion chelation capacity of S100A8/A9 that becomes relevant at high concentrations of S100A8/A9 starting at 20 µg/mL (18, 19).

In this study, we aimed at analyzing whether human BM contains S100-alarmins that contribute to the sepsis-protective effect of BM by providing direct antibacterial effects on pathogens relevant in neonatal sepsis. Accordingly, human BM samples stratified for gestational age, mode of delivery, and sampling after birth were collected at tertiary neonatal care hospitals to determine S100A8/A9 levels and the antimicrobial activity that can be attributed to these proteins.

## Materials and Methods

### Study Approval

The studies were approved by the Institutional Review Board of Hannover Medical School (no. 6143-2012). Mouse experiments were in accordance with German Animal Welfare Legislation and performed as approved by the Lower Saxony State Office for Consumer and Food Safety, Germany (approval no. 33.12-42502-04-14 and no. 33.12-42502-04-15).

### Study Participants

Human BM samples (*n* = 97) were collected from mothers who gave birth to a live baby of 22–41 gestational weeks in the Departments of Obstetrics at the Hannover Medical School, the Henriettenstift Hannover, or the University of Lübeck (Table 1). Written informed consent was obtained from all participating women. Women with chorioamnionitis or systemic infections, major inborn abnormalities, inborn errors of metabolism, and immune-related illnesses were excluded. Mothers were assigned to three groups according to the gestational age of their baby: <32 weeks (extremely and very preterm) (*n* = 41), 32–36 weeks (moderate preterm) (*n* = 37), and ≥37 weeks (term) (*n* = 21). Gestational age was calculated based on the last menstrual period. When early ultrasound at 11–13^+6^ weeks’ gestation using the fetal crown-rump length deviated more than 7 days, dating was performed using ultrasound. Milk was collected on days 1–2 (*n* = 12), 3–5 (*n* = 19), 6–10 (*n* = 35), 11–14 (*n* = 8), 15–30 (*n* = 13), and >30 (*n* = 10) after birth. Milk was expressed by mothers according to their individual feeding protocol either manually or by using a breast pump. Two milliliters of expressed milk were stored at 4°C for a maximum of 12 h. After centrifugation at 1,200 × *g* for 10 min supernatants were stored at −80°C until analyzed for S100A8/A9 concentrations.

**Table 1:** Birth-associated characteristics of the study group providing breast milk samples.

Gestational age	All	<32 weeks	32–36 weeks	≥37 weeks		*p*-Value
Sample size *n* (%)	97 (100)	40 (41)	37 (38)	20 (21)		
Mean gestational age (SD)	33.0 (4.6)	28.6 (2.4)	34.0 (1.2)	39.6 (1.5)		<0.001^a^
Mean age of mothers (SD)	32.3 (6.7)	32.8 (7.3)	32.3 (6.0)	31.1 (6.7)		0.639^a^
Mean parity (SD)	1.7 (1.2)	1.5 (0.8)	1.6 (0.7)	2.2 (2.4)		0.108^a^
Vaginal delivery *n* (%)	33 (34)	11 (27)	11 (30)	11 (55)	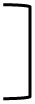	0.090^b^
Cesarean section *n* (%)	64 (66)	29 (73)	26 (70)	9 (45)		

*^a^One-way ANOVA, comparison of all three gestational groups*.

*^b^Fisher’s exact test, comparison of the proportional distribution of delivery modes between all three gestational groups*.

### Reagents

The human S100A8/A9 heterodimer (hS100A8/A9) was isolated from granulocytes as described earlier (22). Human S100A8 and S100A9 were recombinantly expressed in *Escherichia coli* BL21(DE3) as described previously (14, 23). Proteins were analyzed by amino acid sequencing and electrospray ionization mass spectrometry. All preparations revealed >98% purity. Endotoxin contaminations were excluded by limulus amebocyte lysate assay (BioWhittaker), heat inactivation (30 min at 80°C), and polymyxin-B-blocking experiments.

### ELISA Assay

Concentrations of S100A8/A9 in human BM were determined by an in-house ELISA as described previously (14, 24).

### Mice

*s100a9*^−/−^ mice (25) were used for breeding under specific pathogen-free conditions at the Central Animal Facility at the University of Veterinary Medicine Hannover. Enteral administration of S100-alarmins was performed at the age of 1 day by gastric tube feeding. *s100a9^−/−^* pups received 20 µg hS100A8/A9 complex or 5 µg hS100A9 in 20 µL of phosphate-buffered saline (PBS) or aqua alone [control (Ctrl)]. Intraperitoneal injection of hS100A9 and PBS served as positive and negative Ctrls. Plasma was collected 24 h after application of the S100 proteins and immunoblotted against S100A9.

### Bacterial Strains, Media, Growth Conditions

Todd-Hewitt-Bouillon (Roth, Karlsruhe, Germany) was inoculated 1:100 with overnight cultures of *S. aureus* strain Newman (GenBank accession number AP009351.1), *E. coli* (ATCC 25922), and group B *Streptococcus agalactiae* [group B streptococci (GBS), WT 199] and incubated at 37°C with constant shaking at 200 rpm until late-logarithmic growth phase was reached according to optical density at 600 nm (OD_600_), respectively. Bacterial suspensions were concentrated 50 times in succeeding centrifugation steps until the final pellet was resuspended in PBS at a concentration of about 1 × 10^10^ colony-forming units (CFU)/mL.

### Bacterial Growth Inhibition Assays

To test direct antimicrobial activity, S100A8/A9 and S100A8 were diluted in HBSS [without calcium (Ca) and magnesium] at 100 µg/mL while bacterial suspensions of *S. aureus, E. coli* and GBS were diluted 10-fold three times in RPMI medium (Biochrom AG, Berlin, Germany) with 25 mM HEPES. Equal volumes of both preparations were mixed and 200 µL culture aliquots grown at 37°C while shaking at 180 rpm in a 96-well microtiter plate. Bacterial suspensions mixed with HBSS without S100A8 or S100A8/A9 served as Ctrls. Aliquots of the cultures were taken after 0, 2, 5, 8, and 24 h, diluted and plated onto agar plates to determine the number of CFU, respectively. Bacterial growth in the presence of different concentrations of S100A8/A9 with and without the addition of 0.5 mM Ca or in the presence of different concentrations of EDTA and manganese (Mn) was monitored by measuring the increase in OD_600_ over time.

To determine the antimicrobial activity of formula (Humana 0), BM and S100A8/A9-depleted BM, equal volumes were mixed with bacterial suspensions to receive a final concentration of 1 × 10^6^ CFU/mL. Before usage, milk samples were fat reduced by centrifugation at 6,000 × *g* for 10 min. Aliquots of 200 µL from the bacteria–milk suspensions were grown at 37°C while shaking at 180 rpm in a 96-well microtiter plate. Aliquots of the cultures were taken after 0 and 24 h, diluted and plated onto agar plates to determine the number of CFU, respectively.

### Immunoprecipitation (IP) of S100A8/A9 from BM

For the removal of S100A8/A9, BM was first centrifuged at 500 × *g* for 25 min and again at 5,000 × *g* for 30 min. The skimmed supernatant was precleared two times for 1.5 h with Protein A/G Agarose beads (Thermo Scientific, Rockford, IL, USA). Subsequently, the BM was incubated overnight with beads coupled either with the monoclonal antihuman S100A8/A9 antibody (clone 27E10 purified by Vogl) (S100-IP) or a nonspecific polyclonal goat anti-mouse Ctrl antibody (goat anti-mouse IgG-HRP, sc-2005, Santa Cruz Biotechnology, Dallas, TX, USA) (Ctrl-IP). Probes from BM before and after S100-IP and Ctrl-IP as well as from the precipitates were retained for bacterial growth inhibition assays as well as immunoblotting against S100A8.

### Immunoblotting

SDS-PAGE and Western blot staining was performed as described earlier (14, 15, 26) using the primary polyclonal antihuman S100A8 and S100A9 antibody purified by Vogl (14). The anti-rabbit horseradish peroxidase conjugated secondary antibody was obtained from Cell Signaling (Leiden, Netherlands). Protein bands were visualized using the enhanced chemiluminescence system and the ChemiDoc MP System with Image Lab Software v. 4.0 (Bio-Rad Laboratories, Munich, Germany).

### Statistical Analysis

Comparison of S100A8/A9 BM levels between defined groups was performed applying the Mann–Whitney *U*-test. Statistical significance of S100A8/A9 BM levels changing over the time after birth were evaluated by running a Kruskal–Wallis test followed by a *post hoc* Mann–Whitney *U*-test. Comparison of bacterial growth kinetics at defined time points was performed by applying the unpaired two-tailed Student’s *t*-test.

## Results

### S100A8/A9 Levels in BM Are Massively Elevated after Birth

**Figure 1:**
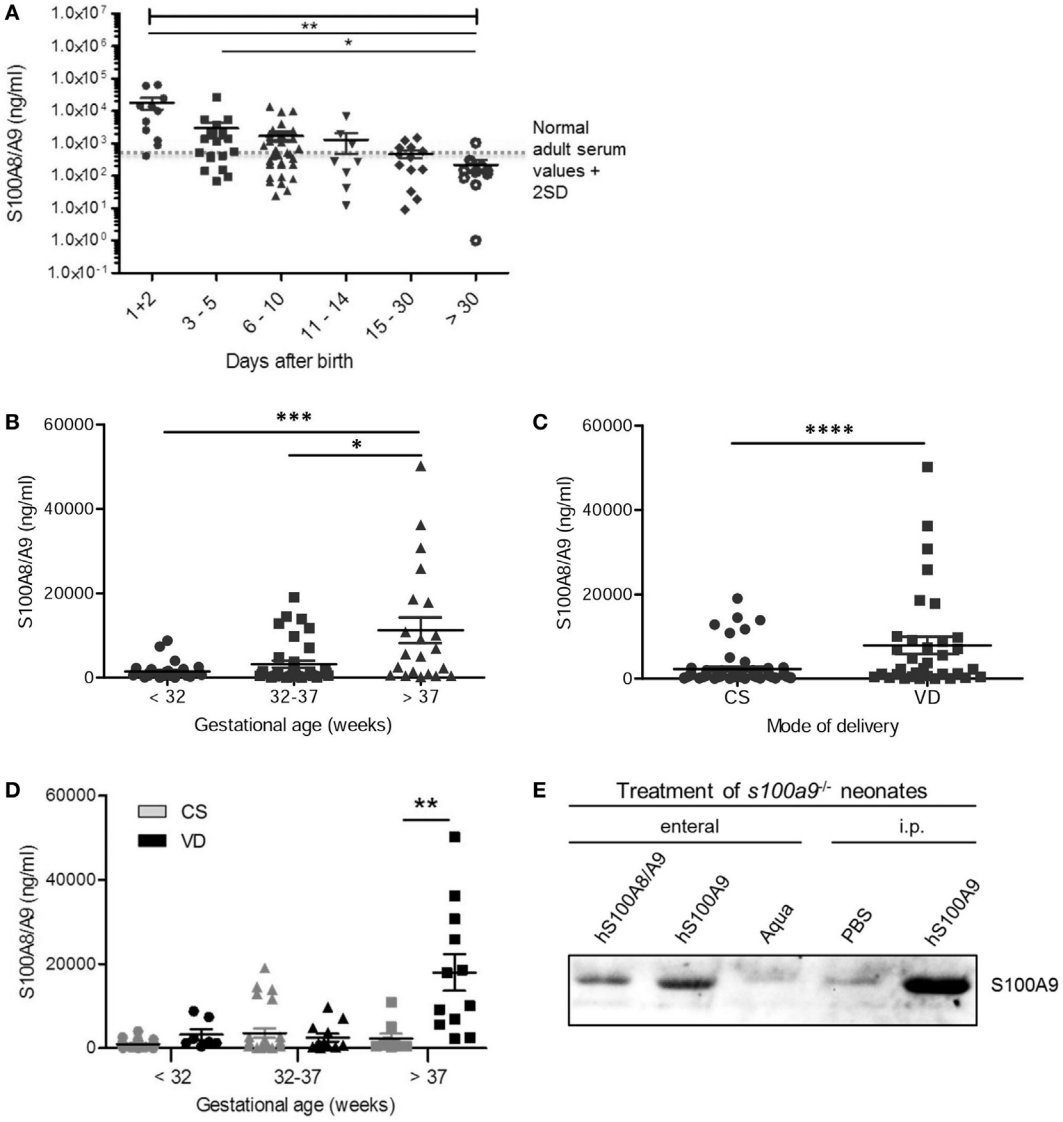
Levels and bioavailability of S100A8/A9 in human breast milk (BM). **(A)** Postnatal course of S100A8/A9 levels in human BM. Bars on a logarithmic scale represent means ± SEM. Significant differences are indicated by capped-end lines across groups (*p* < 0.0005, Kruskal–Wallis test) and open lines (*p < 0.05 and **p < 0.0005, *post hoc* Mann–Whitney *U*-test). **(B–D)** Mean S100A8/A9 levels in human BM during the first 30 days were categorized according to the baby’s gestational age **(B)**, the mode of delivery **(C)**, and the mode of delivery within the different gestational age groups **(D)**. Bars represent means ± SEM. *p < 0.05, **p < 0.01, ***p < 0.005, and ****p < 0.0005, *t*-test. CS, Cesarean section; VD, vaginal delivery. **(E)** S100A9 immunoblotting of plasma from *s100a9^−/−^* neonates collected 24 h after enteral feeding of hS100A8/A9, hS100A9, or aqua. Intraperitoneal (i.p.) injection of hS100A9 and phosphate-buffered saline served as positive and negative control.

Ninety-seven samples of human BM were analyzed and stratified for the day of sampling after birth. During the first 2 days after birth, S100A8/A9 levels in BM were massively elevated (18,400 ± 6,500 ng/mL) compared with normal serum levels in adults (220 ± 40 ng/mL) determined previously (14) (Figure 1A). The levels gradually decreased over time, in average reaching normal adult serum levels 1 month after birth (Figure 1A). S100A8/A9 levels in BM of mothers who gave birth to term babies were significantly higher than of mothers with premature born infants (Figure 1B). Moreover, levels were significantly higher after vaginal delivery (VD) compared with delivery by cesarean section (CS) (Figure 1C). When regarding the gestational groups separately, significant dependence on the mode of delivery was only evident after term delivery. Within both preterm groups, the differences between VD and CS were not significant (Figure 1D).

To provide first evidence that enterally supplied S100-alarmins are bioavailable for the newborn, we used *s100a9^−/−^* mice that were fed with 20 µg S100A8/A9 or 5 µg S100A9 on day 1 of life. Twenty-four hours after feeding, the alarmins could be retrieved systemically in the plasma of these mice (Figure 1E). Collectively, the findings demonstrated that BM supplies newborn infants with high amounts of bioavailable S100-alarmins after birth, especially, when they are born at term and delivered vaginally.

### S100A8/A9 Acts Bacteriostatic against Important Pathogens of Neonatal Sepsis

**Figure 2:**
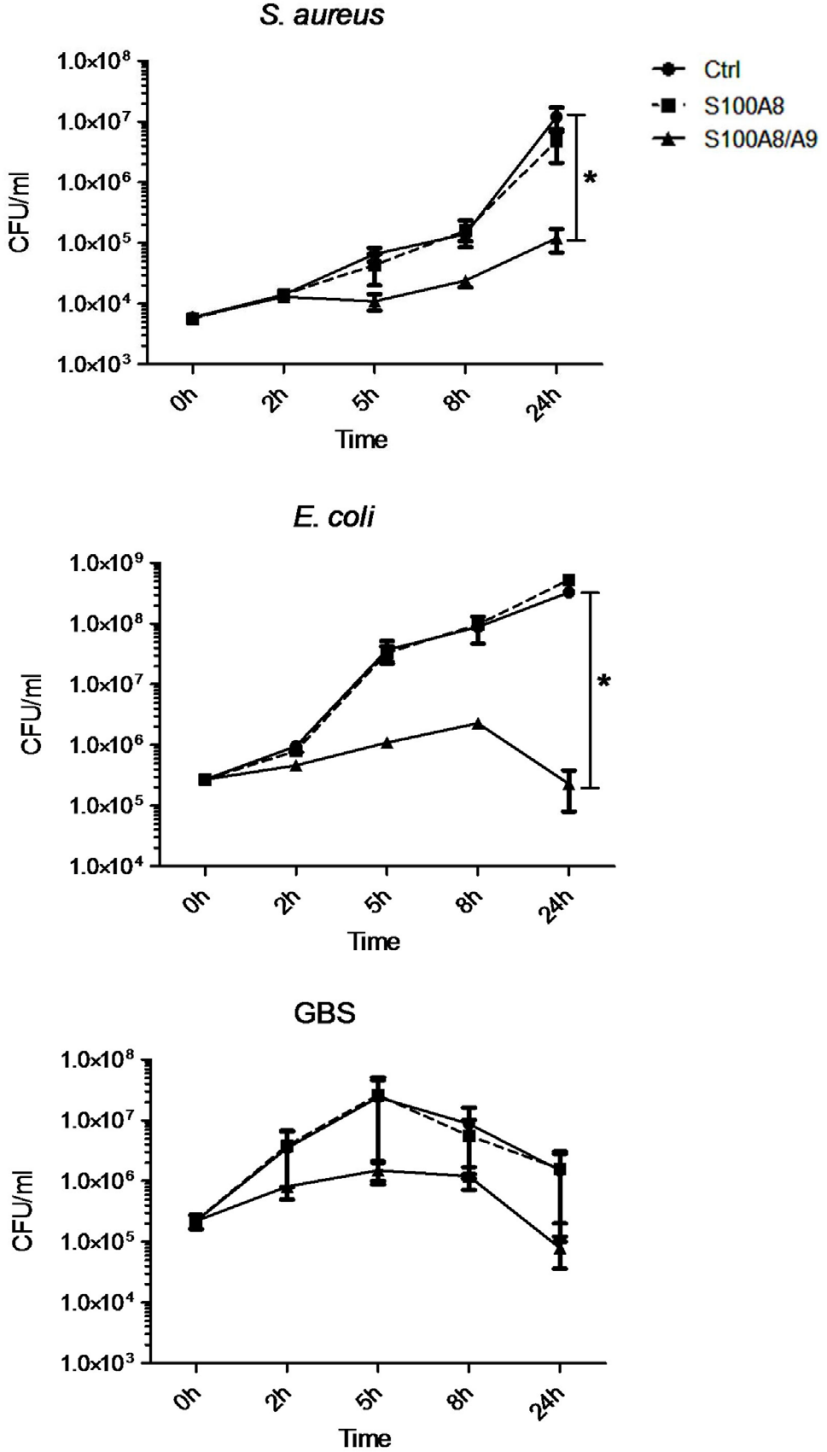
S100A8/A9 inhibits the growth of pathogens causing neonatal sepsis. *Staphylococcus aureus, Escherichia coli*, and group B streptococci (GBS) were grown in the absence [control (Ctrl)] and presence of 50 µg/mL S100A8/A9 or S100A8. The concentration of colony-forming units (CFU) over culture time was plotted as mean ± SEM. *p < 0.05, *t*-test (*n* = 4).

**Figure 3:**
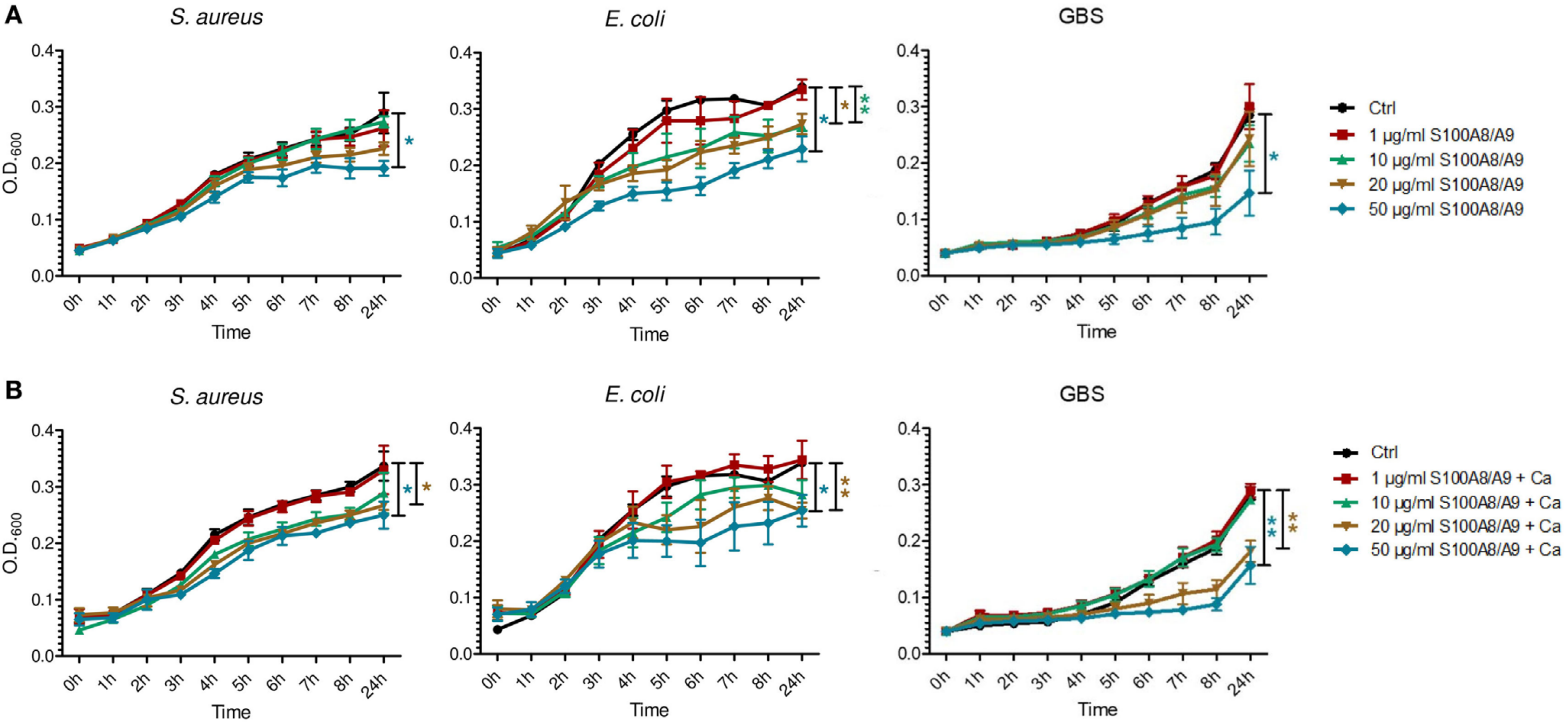
S100A8/A9 exerts antimicrobial activity at concentrations detectable in breast milk. *Staphylococcus aureus, Escherichia coli*, and group B streptococci (GBS) were grown at indicated concentrations of S100A8/A9 in the absence **(A)** and presence of 0.5 mM Ca **(B)**. OD_600_ values over time were plotted as mean ± SEM. *p < 0.05 and **p < 0.05, *t*-test (*n* = 3).

To clarify the antimicrobial role of S100 proteins in BM, we assessed the activity of S100A8/A9 and S100A8 on the growth of three of the most common pathogens causing neonatal sepsis. The heterodimer complex S100A8/A9 dose-dependently inhibited the growth of *S. aureus, E. coli*, and GBS (Figures 2 and 3), while the homodimer S100A8 exerted no antimicrobial activity (Figure 2), which was in accordance with previous reports (18). In the absence of Ca, bacteriostatic activity against *S. aureus* and GBS required the presence of high concentrations of S100A8/A9 (50 µg/mL) (Figure 3A). However, in the presence of Ca, which is present in high amounts in BM, the growth inhibitory activity of S100A8/A9 was significantly enhanced; then S100A8/A9 concentrations of 10 µg/mL against *S. aureus* and 20 µg/mL against GBS were sufficient for significant growth inhibition (Figure 3B). Impairment of *E. coli* growth also started at concentrations of 10–20 µg/mL S100A8/A9, but was independent of the presence of Ca (Figures 3A,B).

### S100A8/A9 Contributes to the Antimicrobial Activity of BM Targeting Mn-Sensitive Pathogens

**Figure 4:**
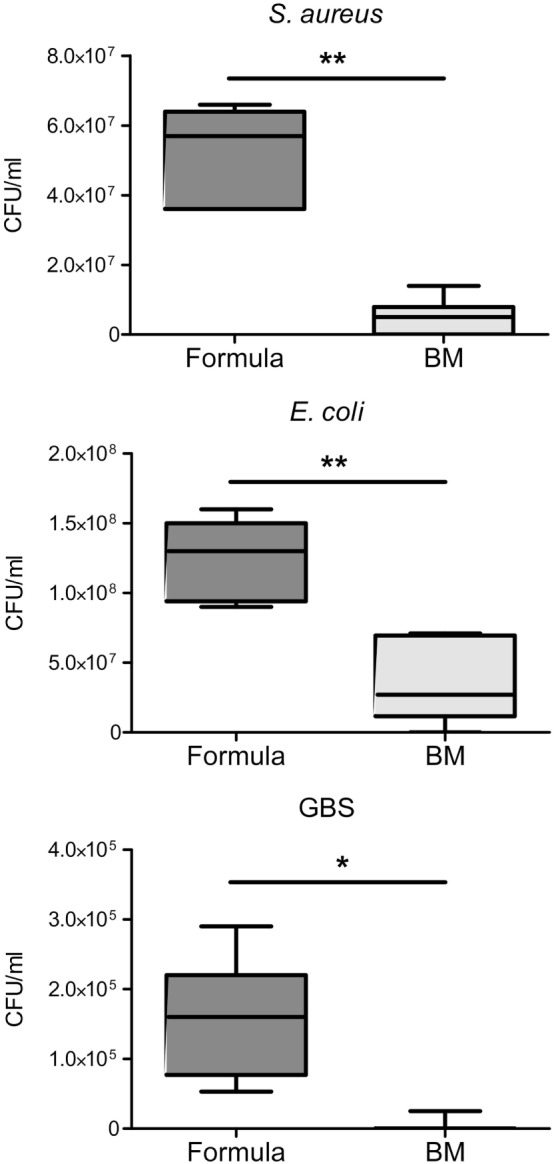
Breast milk (BM) inhibits the growth of sepsis-causing pathogens. *Staphylococcus aureus, Escherichia coli*, and group B streptococci (GBS) were grown over 24 h in infant formula and BM. Box plots show medians (center lines) and interquartile ranges of the concentration of colony-forming units (CFU) ± SEM. *p < 0.005 and **p < 0.00005, *t*-test.

**Figure 5:**
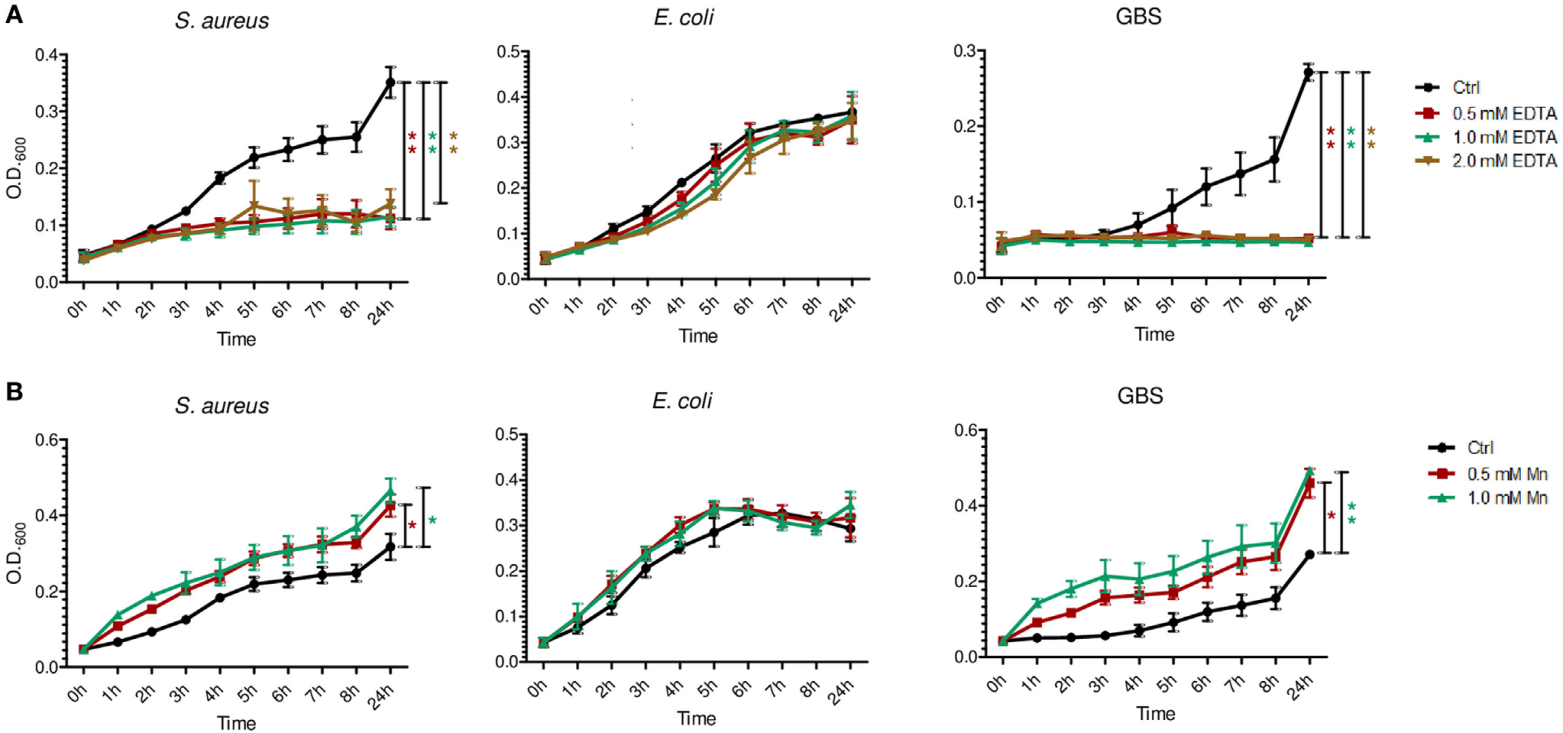
Differential susceptibility of bacterial growth to metal ion depletion. *Staphylococcus aureus, Escherichia coli*, and group B streptococci (GBS) were grown at indicated concentrations of EDTA **(A)** and manganese **(B)**. OD_600_ values over time were plotted as mean ± SEM. *p < 0.05 and **p < 0.001, *t*-test (*n* = 3).

In line with previous reports of BM effects on *S. aureus* and *E. coli* (27, 28), we found the growth of *S. aureus, E. coli*, and also GBS in BM significantly reduced compared with their growth in formula (Figure 4). To investigate whether and to what extent the presence of S100A8/A9 in BM is responsible for these effects we first elucidated what bacteriostatic mechanism of S100A8/A9 might be relevant in BM and for which of the bacterial strains. Chelation of divalent metal ions like Mn, zinc and iron has been reported to be the main mechanism of antimicrobial activity of S100A8/A9 (18, 19, 29, 30). Therefore, we first assessed the general susceptibility of the bacterial strains to metal ion depletion. The growth of *S. aureus* and GBS, but not of *E. coli*, was strongly impaired in the presence of EDTA (Figure 5A). Considering the molar ratios and binding capacities of S100A8/A9 for divalent ions, Mn depletion is the most likely antimicrobial mechanism of S100A8/A9 relevant in BM. A concentration of 50 µg/mL S100A8/A9 corresponds to 1 µM S100A8/A9. Comparatively, zinc and iron are in excess in BM (about 0.1–0.3 mM). However, the average content of Mn in BM is only about 2 µM. Therefore, we tested the sensitivity of bacterial growth toward Mn supplementation. In contrast to *E. coli*, the growth of *S. aureus* and GBS was strongly promoted by Mn (Figure 5B), which was in line with the resistance of *E. coli* against divalent ion depletion by EDTA (Figure 5A). The data suggested that S100A8/A9-mediated antimicrobial activity of BM primarily might be relevant for Mn-sensitive strains like *S. aureus* and GBS, but less important for largely Mn-insensitive bacteria like *E. coli*.

**Figure 6:**
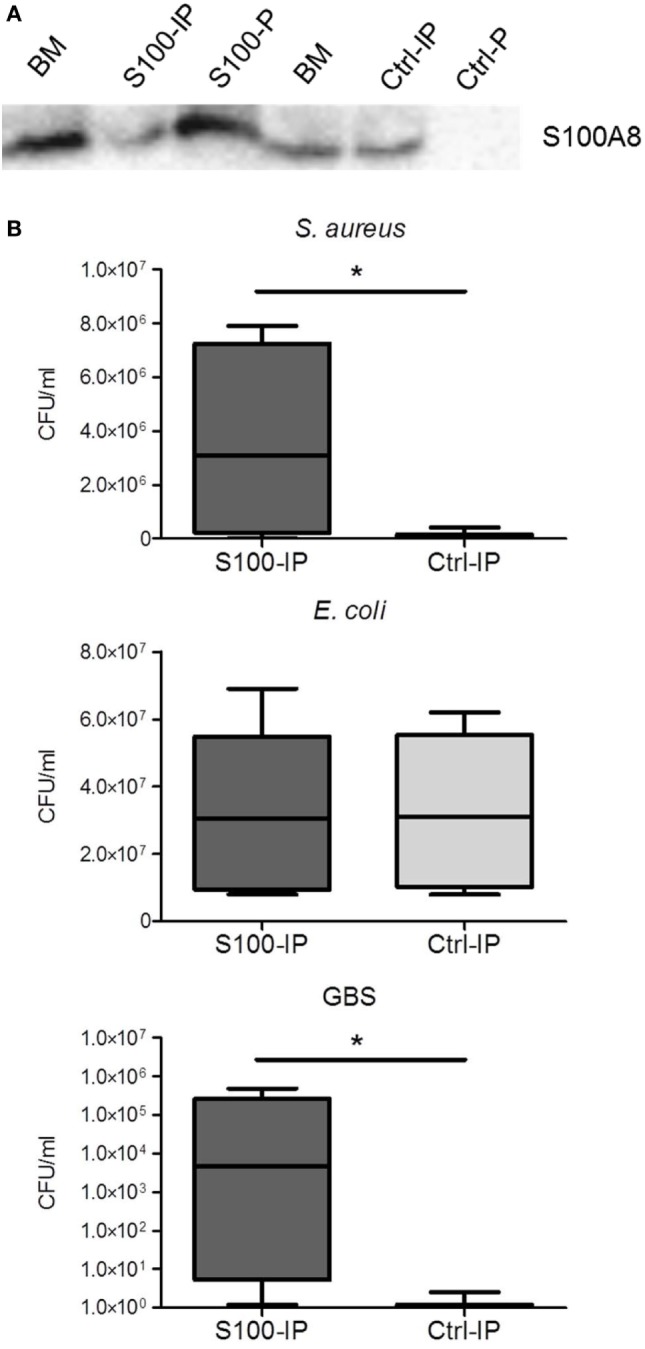
Depletion of S100A8/A9 decreases the antimicrobial activity of breast milk (BM). **(A)** Efficiency of S100A8/A9 removal was determined by immunoblotting against S100A8. Representative blot showing BM probes before and after S100-IP and control (Ctrl)-IP as well as the precipitates (S100-P and Ctrl-P). **(B)** *Staphylococcus aureus, Escherichia coli*, and group B streptococci (GBS) were grown over 24 h in S100A8/A9-depleted (S100-IP) and non-depleted (Ctrl-IP) BM samples. Box plots show medians (center lines) and interquartile ranges of the concentration of colony-forming units (CFU) ± SEM. *p < 0.05, *t*-test.

To validate this hypothesis, we removed S100A8/A9 from BM by IP (Figure 6A). The bacteriostatic effect of BM against *S. aureus* and GBS was significantly reduced after depletion of S100A8/A9 (Figure 6B). However, *E. coli* grew equally in unmodified and S100A8/A9-depleted BM (Figure 6B). Collectively, our data demonstrate that S100A8/A9 significantly contributes to the antimicrobial activity of BM against highly relevant, especially Mn-sensitive, pathogens of sepsis in newborns.

## Discussion

More than 400,000 babies die of neonatal sepsis per year worldwide making it one of the most common reasons for death in the neonatal period (9). Breast feeding reduces neonatal morbidity and mortality by infectious diseases as sepsis resulting in relevant cost savings to health-care providers (3, 10–12, 31–34). The molecular mechanisms how BM achieves these sepsis-protective effects are not yet completely elucidated. It is unquestionable that antimicrobial effects of BM contribute to the protection from infections and sepsis. Direct growth inhibition of *S. aureus* and *E. coli* by BM has been demonstrated, but the molecular mechanism remained unclear; bacteriostatic effects were only correlated with levels of antimicrobial proteins in BM, correlating best with lactoferrin (27, 28). However, until now, most efforts of nutritional supplementation of formula with bioactive components from human BM remained without success in the clinical setting and did not decrease the incidence of sepsis (2, 13). Next to antimicrobial activity, immunoregulatory effects of BM might be even more relevant with respect to sepsis protection since neonates are primed to respond hyperinflammatory upon microbial challenges ending up with rapid fatal courses of sepsis (6, 14–16). However, mediators and molecular mechanisms of immunoregulation by BM are still broadly elusive.

In this study, we hypothesized that S100-alarmins might be good bioactive candidate components that unify immunoregulatory (14–16) and antimicrobial (18, 19) properties of BM. For the first time, we showed that S100A8/A9 levels in BM after birth were at least six times higher than the already elevated S100A8/A9 serum levels in healthy term newborns. Similar to the postnatal course of S100A8/A9 serum levels of the descendants (14), S100A8/A9 concentrations in BM decreased over the first few weeks after birth to normal adult serum levels. They were highest after term compared with preterm delivery and after VD compared with CS, both again comparable to conditions of S100A8/A9 serum levels in babies born term versus preterm (15) or born per VD compared with CS (unpublished data). Our findings point to a closely stress-associated release of S100A8/A9 into BM during the process of birth. Whereas S100A8/A9 BM levels of mothers after term delivery were strictly dependent on the mode of delivery, this was only a trend after extremely or very preterm delivery and did not hold true for the group of mothers after moderate preterm birth. A good explanation for the latter might be the high proportion of secondary CS (31%) in the moderate preterm group. Secondary CS implicates a varying degree of contractions and labor that might be comparable to labor during VD leading to a stress-associated release of S100A8/A9 into the BM.

With respect to antimicrobial properties, it has previously been shown that S100A8/A9 only acts bacteriostatic at high concentrations starting at 20 µg/mL (18, 19). BM contains high amounts of Ca. We could show that in the presence of Ca antimicrobial activity becomes significant at 10 µg/mL, i.e., at average concentrations detectable in human BM. At such high concentrations, depletion of divalent metal ions by S100A8/A9 was shown to become relevant hampering bacterial growth (18, 19, 29). In line with others (27, 28), we demonstrated that BM inhibited the growth of *S. aureus, E. coli*, and GBS, three of the most common causal pathogens of sepsis in newborns (7, 35–39). In this study, we could clearly show that S100A8/A9 in BM contributes significantly to the bacteriostatic activity of BM on *S. aureus* and GBS as depletion of S100A8/A9 from BM enhanced the growth of these strains. However, the growth of *E. coli* in BM remained unaffected after removal of S100A8/A9. The reason is most likely the demonstrated independence of *E. coli* on the presence of Mn in contrast to the high Mn-sensitivity of *S. aureus* and GBS growth. BM contains only trace amounts of Mn (about 2 µM) compared with an excess of Zn and iron (0.1–0.3 mM). Considering the chelating capacities of the highest S100A8/A9 levels (1–2 µM) detectable in BM, Mn depletion is certainly the most relevant antimicrobial mechanism of S100A8/A9 in BM. Consequently, the growth inhibitory effect of BM against *E. coli* must rather be mediated by other antimicrobial factors than S100A8/A9 present in BM.

Interestingly, with respect to immunoregulatory effects, the homodimer S100A8 has been demonstrated to be more potent in neonates than the heterodimer complex of S100A8/A9 (15, 16). However, in contrast to S100A8/A9, S100A8 did not inhibit the growth of any of the tested pathogens of neonatal sepsis (*S. aureus, E. coli*, and GBS), which is most likely due to the lack of transitional binding sites for divalent metal ions (29). These data are in accordance with findings of others on the antimicrobial activity of S100-alarmins on *S. aureus* and *K. pneumoniae* (18, 19).

The high amount of S100-alarmins in BM next to their massive systemic release by the newborn itself suggests that the supply of newborns with S100-alarmins is of vital importance. We previously showed that the release of S100-alarmins is an essential mechanism in newborn infants that prevents excessive inflammation and fatal septic courses by inducing a state of inflammatory hyporesponsiveness (14–16). In this study, we demonstrated in murine *s100a9^−/−^* neonates that enterally supplied S100-alarmins can even be detected systemically in the plasma of fed mice. Immunoregulation by S100-alarmins is already active at serum concentrations one log level lower than the S100A8/A9 levels in BM. So, the biological relevance of S100A8/A9 in BM seems to be double. Due to their high concentrations in BM antimicrobial and immunoregulatory properties, both might contribute to the sepsis-protective effect of BM. Interestingly, Lee et al. demonstrated that early feeding with colostrum significantly reduces clinical sepsis in extremely low birth weight infants (40). We found BM of the first 2 days after delivery including colostrum to be massively loaded with S100-alarmins.

Next to the importance for the newborn infant, antimicrobially active amounts of S100A8/A9 in BM might also be important for the mother by protecting from lactational mastitis, especially given the fact that *Staphylococci* and *Streptococci* are the most common pathogens found in mastitis (41, 42).

Our investigations demonstrate that BM contains vast amounts of S100-alarmins. Levels are highest in the group of newborns with the lowest sepsis risk, namely, term infants delivered vaginally. Concentrations are so high that next to immunoregulatory functions S100A8/A9 in BM acts antimicrobially against important pathogens causing neonatal sepsis. These findings point to S100-alarmins as attractive bioactive components whose enteral supply could prevent fatal courses in newborns that are at high risk to suffer from sepsis and inflammation.

## Ethics Statement

The studies were approved by the Institutional Review Board of Hannover Medical School (no. 6143-2012). Written informed consent was obtained from all participating women. Mouse experiments were in accordance with German Animal Welfare Legislation and performed as approved by the Lower Saxony State Office for Consumer and Food Safety, Germany (approval no. 33.12-42502-04-14 and no. 33.12-42502-04-15).

## Author Contributions

SP and DV conceptualized and designed the study; SP, MR, JP, and CH collected samples and data; MR, BF, and TV performed experiments; SP, CH, JR, TV, and DV analyzed the data; SP and MR drafted the initial manuscript; and SP, MR, CH, JR, TV, and DV reviewed and revised the manuscript. All the authors approved the final manuscript as submitted and agreed to be accountable for all aspects of the work.

## Conflict of Interest Statement

The authors declare that the research was conducted in the absence of any commercial or financial relationships that could be construed as a potential conflict of interest.

## Abbreviations

BM, breast milk; CFU, colony-forming units; CS, cesarian section; *E. coli, Escherichia coli*; GBS, group B streptococci; IP, immunoprecipitation; *S. aureus, Staphylococcus aureus*; TLR4, toll-like receptor 4; VD, vaginal delivery.
